# Heat Shock Protein 60 in Cardiovascular Physiology and Diseases

**DOI:** 10.3389/fmolb.2020.00073

**Published:** 2020-04-30

**Authors:** Yaoyun Duan, Huayuan Tang, Kali Mitchell-silbaugh, Xi Fang, Zhen Han, Kunfu Ouyang

**Affiliations:** ^1^State Key Laboratory of Chemical Oncogenomics, School of Chemical Biology and Biotechnology, Peking University Shenzhen Graduate School, Shenzhen, China; ^2^Department of Medicine, School of Medicine, University of California, San Diego, La Jolla, CA, United States; ^3^Department of Cardiovascular Surgery, Peking University Shenzhen Hospital, Shenzhen, China

**Keywords:** heat shock protein, HSP60, heart failure, atherosclerosis, cardiomyocyte

## Abstract

Heat shock protein 60 (HSP60) is a highly conserved protein abundantly expressed in both prokaryotic and eukaryotic cells. In mammals, HSP60 has been primarily considered to reside in the mitochondria, where HSP60 and HSP10 form a complex and facilitate mitochondrial protein folding. However, HSP60 is also observed in the cytoplasm, the plasma membrane, and the extracellular space. HSP60 regulates a broad spectrum of cellular events including protein trafficking, peptide hormone signaling, cell survival, cell proliferation, inflammation, and immunization. In the cardiovascular system, growing evidence indicates that HSP60 could not only play an important role under physiological conditions, but also regulate the initiation and progression of heart failure and atherosclerosis. In this review, we focus on recent progress in understanding the function of HSP60 in cardiomyocytes, endothelial cells, and vascular smooth muscle cells (VSMCs), respectively, and discuss the related signaling pathways that have been found in these cells, so as to illustrate the role of HSP60 in the development of cardiovascular disease.

## Introduction

The human heat shock protein 60 (HSP60), which is also known as 60 kDa chaperonin, belongs to a family of the most ancient and conserved proteins in both prokaryotic and eukaryotic cells. They have a high homology between species and are ubiquitously expressed in most cells. HSP60 was initially found as a mitochondrial protein that plays a critical role in regulating mitochondrial protein homeostasis (Cheng et al., 1989; Ostermann et al., 1989). It has been shown that human HSP60 and HSP10 form a symmetrical football complex (Nisemblat et al., 2015), whereas the bacteria homolog of HSP60, GroEL, is organized in two rings, producing a barrel-like structure (Ostermann et al., 1989; Horwich et al., 2006). A survey of interactors of the human HSP60 suggested that most HSP60-interacting proteins are localized to the mitochondrial matrix space and involved in various mitochondrial functions and metabolic pathways (Bie et al., 2020). Interestingly, accumulating studies have demonstrated that HSP60 is also localized in extramitochondrial compartments including the cytosol, plasma membrane, and extracellular space, as well as in blood circulation (Meng et al., 2018). Depending on protein localization, HSP60 not only regulates the mitochondrial chaperoning activity, but also plays a functional role in multiple cellular processes including cell proliferation, apoptosis, migration, and immune responses (Henderson et al., 2013).

The cardiovascular system is comprised of the heart and the network of arteries, veins, and capillaries that transport blood throughout the body. In humans, cardiovascular disease is a leading cause of mortality throughout the world (Go et al., 2014). It has been shown that many risk factors for cardiovascular disease, including smoking, lipopolysaccharide, chlamydia pneumoniae, shear stress, and ischemia, can promote the expression of HSP60 (Jakic et al., 2017). In the cardiovascular system, HSP60 has been supposed to play a regulatory role in cardiomyocytes, endothelial cells (ECs), vascular smooth muscle cells (VSMCs), and immune cells under both physiological and pathological conditions such as heart failure and atherosclerosis. The functions of HSP60 in immune cells and immune regulation have been well addressed and discussed elsewhere (Grundtman et al., 2011; Quintana and Cohen, 2011; Wick et al., 2014; Zininga et al., 2018). This review focuses on the functional roles that HSP60 performs in cardiomyocytes, ECs, and VSMCs, respectively, and the involvement of HSP60 in the pathogenesis of heart failure and atherosclerosis.

## Cardiac HSP60 and Heart Failure

**FIGURE 1:**
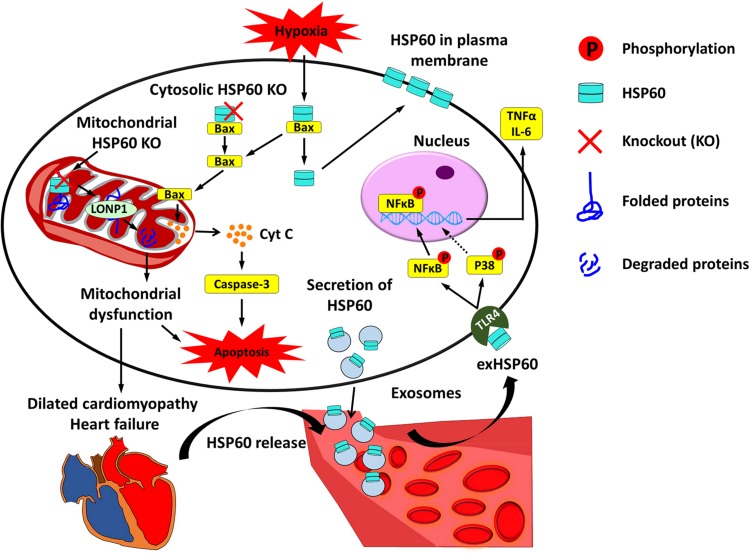
Cardiac HSP60 and heart failure. In cardiac cells, HSP60 is located on the membrane and in the mitochondria, cytoplasm, and extracellular space. Mitochondrial HSP60 facilitates the folding of mitochondrial proteins and prevents mitochondrial protein degradation. HSP60 deletion in adult mouse hearts impels HSP60-dependent mitochondrial proteins to undergo degradation via LONP1 and causes mitochondrial dysfunction, which eventually leads to dilated cardiomyopathy and heart failure. Cytosolic HSP60 is co-localized with Bax and plays an anti-apoptotic role in cardiac cells. Loss of cytosolic HSP60 causes translocation of Bax to the mitochondria, release of Cytochrome C (Cyt C), activation of Caspase-3, and apoptosis. In addition, hypoxia triggers apoptosis via inducing the disassociation of the HSP60-Bax complex by translocating cytosolic HSP60 to the plasma membrane and Bax to the mitochondria. Extracellular HSP60 (exHSP60) can be released by cardiomyocytes via exosomes or other damaged cells. It binds to Toll-like receptor4 (TLR4) and induces the release of tumor necrosis factor α (TNFα) and Interleukin 6 (IL-6) via activation of NFκB and JNK. In heart failure, HSP60 is released from cardiomyocytes. Increased serum levels of HSP60 are related to the severity of heart failure.

HSP60 is highly expressed in cardiac tissues, and has been found in different subcellular locations inside cardiomyocytes, including on the membrane and in the mitochondria, cytoplasm, and extracellular space (Gupta and Knowlton, 2007; Lin et al., 2007). A large number of studies using *in vitro* cell culture models and *in vivo* animal models have revealed that HSP60 plays an important role in regulating cardiac physiology and pathophysiology. Here we describe several major roles of HSP60 in cardiomyocytes and the involvement of HSP60 in the progression of heart failure (Figure 1).

### Intracellular HSP60 May Play Protective Roles in Cardiac Cells

In cardiac cells, HSP60 is mainly located inside the mitochondria, while a small portion of HSP60 (approximately 20–40%) can be observed in the cytoplasm (Lin et al., 2007). In cultured neonatal rat cardiomyocytes, overexpression of HSP60 alone or together with its co-chaperone HSP10 protected myocytes against apoptosis induced by simulated ischemia and reoxygenation (Lau et al., 1997; Lin et al., 2001). The protective function of overexpressed HSP60 and HSP10 is associated with reduced mitochondrial Cytochrome c release and suppressed Caspase-3 activity, as well as an increase of ATP recovery and elevated activities of mitochondrial complexes III and IV. These results suggest that mitochondrial chaperonin HSP60 plays a critical role in regulating mitochondrial integrity and capacity for ATP production, which are essential for determining the survival of cardiomyocytes undergoing ischemia and reperfusion injury.

Human mitochondrial HSP60 and its cochaperonin HSP10 form a symmetrical football complex (Nisemblat et al., 2015), which facilitates the folding of mitochondrial proteins and therefore confers their stability. This consequently prevents mitochondrial protein degradation and the induction of mitochondrial unfolded protein responses. Furthermore, HSP60 may protect mitochondrial proteins from aggregation, especially under stressful conditions. Myrtucommulone, a natural product that can inhibit the refolding activity of the HSP60/HSP10 complex, prevents the reactivation of denatured malate dehydrogenase in a protein refolding assay. Under heat shock, the interference of myrtucommulone with HSP60 is accompanied by aggregation of the Lon protease-like protein (LONP) and the leucine-rich PPR motif-containing protein (LRP130) (Wiechmann et al., 2017; Meng et al., 2018). In future studies, it will be very interesting to examine whether HSP60 deletion in cardiac cells could also result in the aggregation of certain mitochondrial proteins under stress.

HSP60 is also observed in the cytosol, where it may exist in monomeric or heptametric forms (Taguchi et al., 1994; Levy-Rimler et al., 2001). HSP60 is synthesized in the cytosol with a mitochondrial transport signal (Singh et al., 1990). After translocation to the mitochondria, the mitochondrial transport signal is then cleaved and a certain amount of HSP60 protein may return to the cytosol. On the other hand, HSP60 with the mitochondrial target peptide may also be observed in the cytosol as the newly synthesized protein and accumulation of such proteins could be found in certain circumstance without apparent mitochondrial release (Chandra et al., 2007). However, the mechanism underlying the distribution of HSP60 between the mitochondria and the cytosol remains unclear. HSP60 in the cytosol has also been considered to play an antiapoptotic role in cardiac cells. Immuno-electron microscopy has demonstrated that HSP60 is co-localized with Bax in the cytosol of normal rat hearts (Gupta and Knowlton, 2005). The decrease of cytosolic HSP60 induced by an antisense phosphorothioate oligonucleotide facilitates the translocation of Bax to the mitochondria and induces apoptosis, evidenced by the release of mitochondrial Cytochrome c, activation of Caspase 3, and induction of DNA fragmentation (Kirchhoff et al., 2002). Moreover, the hypoxia can trigger the disassociation of the HSP60-Bax complex, accompanied with the translocation of Bax to the mitochondria and cytosolic HSP60 to the plasma membrane, which may be sufficient to induce cell apoptosis in adult rat cardiomyocytes (Gupta and Knowlton, 2002; Knowlton and Gupta, 2003).

### HSP60 Is Essential for Maintaining Mitochondrial Function and Cardiac Physiology

The importance of HSP60 has been characterized in *Escherichia coli* and yeast, in which the deficiency of HSP60 leads to a lethal phenotype (Cheng et al., 1989; Fayet et al., 1989). HSP60 is also essential for survival of Drosophila and mice (Perezgasga et al., 1999; Christensen et al., 2010). Inactivation of the *Hspd1* gene in mice results in early embryonic lethality while *Hspd1* haploinsufficiency can also cause a late onset and slowly progressive deficit in motor functions (Christensen et al., 2010; Magnoni et al., 2013). Moreover, it has been shown that missense mutations in human HSPD1 gene are associated with two extremely rare monogenic disorders-hereditary spastic paraplegia and a recessively inherited white matter disorder (Hansen et al., 2002; Magen et al., 2008). Recently, we generated a mouse model with inducible cardiac-specific HSP60 deletion and investigated the role of HSP60 in regulating mitochondrial function and cardiac physiology (Fan et al., 2019). We found that deletion of HSP60 in adult cardiomyocytes dramatically altered the activities of mitochondrial complexes, reduced mitochondrial membrane potential, increased ROS production, and eventually resulted in dilated cardiomyopathy, heart failure, and death of mice. Proteomic analysis in purified HSP60-deficient mitochondria suggested that about 20% of mitochondrial-localized proteins are HSP60-dependent, meaning they rely on HSP60 to mediate correct protein folding in the mitochondria. A survey of HSP60-interacting proteins was recently performed in HEK293 cells using co-immunoprecipitation and mass spectrometry and identified more than 300 proteins (Bie et al., 2020), 46 of which including ALDH2, CPT2, IDH3A, and SUCLG2 were downregulated in mitochondria of HSP60-deficient cardiomyocytes (Fan et al., 2019). Furthermore, an assessment of the mitochondrial protein import and stability found that deletion of HSP60 has no effect on mitochondrial protein import. However, HSP60 deletion impels HSP60-dependent mitochondrial proteins to undergo degradation after import, which suggests that the protein exhibits low stability in HSP60-deficient mitochondria. Moreover, deletion of HSP60 activates the mitochondrial unfolded protein response (Fan et al., 2019) and is also accompanied with increased cell apoptosis (Fan et al., 2019). All these results together demonstrate that HSP60 is required for maintaining normal mitochondrial function and cardiac physiology.

### Extracellular HSP60 May Have an Injurious Effect on Cardiomyocytes

It is now clear that HSP60 also exists in the plasma, as well as in the extracellular space of cardiomyocytes (Pockley et al., 2000; Lewthwaite et al., 2002; Giannessi et al., 2007; Gupta and Knowlton, 2007; Lin et al., 2007; Kim et al., 2009; Li et al., 2011; Blasi et al., 2012), even though the exact mechanism underlying how HSP60 is secreted from cardiomyocytes is still under debate. First, HSP60 can be passively released as the intact or fragmented protein from damaged or dead cells, respectively (Basu et al., 2000). Secondly, HSP60 in certain non-cardiomyocytes can be secreted through the conventional endoplasmic reticulum–Golgi secretory pathway (Hayoun et al., 2012; Campanella et al., 2016). However, a nonconventional secretion mechanism, the lipid raft-exosome pathway, is a more widely accepted way by which HSP60 in cardiomyocytes is secreted (Gupta and Knowlton, 2007; Lin et al., 2007). HSP60 in the exosomes is found to bind with the exosome membrane, and is released via exosomes in both the basal state and subsequent mild stress (Gupta and Knowlton, 2007; Lin et al., 2007). HSP60 can be used as a marker to indicate the number of extracellular vesicles released from the heart (Giricz et al., 2014). Alternatively, another point of view suggests that HSP60 may be stabilized within the exosome under multiple certain physiological conditions and is not released to prevent its toxicity to cardiomyocytes (Malik et al., 2013).

It is worthy to note that HSP60 in the plasma/serum reflects the total amount of the protein released by all types of organs and tissues, and extracellular HSP60 (exHSP60) in the heart tissue can be released by cardiomyocytes via exosomes as well as by necrosis or other routes, as mentioned above. In any case, exHSP60 is generally considered as an injurious signal in cardiomyocytes (Kim et al., 2009; Tian et al., 2013), even though exHSP60 has also been shown to play a beneficial effect on certain non-cardiomyocytes like B cells (Cohen-Sfady et al., 2009). exHSP60 may function as a ligand for the Toll-like receptors (TLRs) in many cell types including cardiomyocytes. TLR4 is the most highly expressed subtype of TLRs in cardiomyocytes (Frantz et al., 1999; Heiserman et al., 2015), and has been considered as the receptor of exHSP60 (Ohashi et al., 2000; Kim et al., 2009; Tian et al., 2013). exHSP60 can induce the release of tumor necrosis factor a (TNFα) and Interleukin 6 from cardiomyocytes, which can be impeded by the inhibitors of P38 and NFκB (Tian et al., 2013). Meanwhile, exHSP60 also increases the expression of TLR2 and TLR4 in cardiomyocytes, which can be abolished by the inhibitors of JNK and NFκB (Tian et al., 2013). Activation of NFκB eventually leads to the release of Cytochrome C and AIF from the mitochondria, activation of Caspase-3/7, and cell apoptosis in cardiomyocytes treated with HSP60 (Kim et al., 2009; Knowlton, 2017). Consistently, treatment with an anti-TLR4 blocking antibody or deletion of TLR4 is able to totally abolish the exHSP60-induced cell apoptosis in cardiomyocytes (Kim et al., 2009; Heiserman et al., 2015). In addition, exHSP60 released from cardiomyocytes via exosomes or in other ways may also play a role in regulating cardiac fibroblast and endothelial cell functions, and thus affects the adaptive responses of hearts under stress (Cervio et al., 2015).

### HSP60 May Act as a Biomarker of Heart Failure

Heart failure, a process of chronic inflammation and progressive injury of cardiac muscle, is one of the most common complications of cardiovascular disease (Rizzo et al., 2011). Heart failure can result from many forms of heart disease, including dilated and ischemic cardiomyopathy. It has been shown that the expression of endogenous HSP60 is significantly elevated in the myocardium of patients with dilated and ischemic cardiomyopathy (Knowlton et al., 1998; Latif et al., 1999). During the progression of heart failure, NFκB is chronically activated, resulting in increased binding to the two NFκB binding elements in the HSP60 gene. This may account for increased expression of HSP60 in cardiomyocytes (Wong et al., 1998; Wang et al., 2010). In addition to the overall change of the HSP60 protein, HSP60 may redistribute between different subcellular locations in the cardiomyocyte under stress. As mentioned above, HSP60 in the cytosol can be translocated to the plasma membrane, which may cause the movement of Bax to the mitochondria as well as the activation of Caspases and apoptosis in the cardiomyocytes during heart failure (Gupta and Knowlton, 2007). This is consistent with a finding that HSP60 levels in the cytosol is reduced in dilated cardiomyopathy (DCM) hearts (Sidorik et al., 2005).

More importantly, serum HSP60 (sHSP60) may act as a biomarker for heart failure. Detectable sHSP60 levels have been observed in both healthy control human patients (Lewthwaite et al., 2002; Halcox et al., 2005) and patients with cardiovascular diseases (Xu et al., 2000; Shamaei-Tousi et al., 2006, 2007; Zhang et al., 2008). Acute myocardial infarction is able to induce HSP60 release, as manifested by a rise of sHSP60 levels soon after the onset of acute myocardial infarction and a positive correlation of these levels. Acute myocardial infarction also leads to adverse cardiovascular events and increased levels of creatine phosphokinase and troponin (Zhang et al., 2008; Novo et al., 2011). In patients with advanced chronic heart failure secondary to ischemic or idiopathic dilated cardiomyopathy, sHSP60 is correlated to the severity of the disease and is associated with a high risk of adverse cardiac events (Niizeki et al., 2008). In patients with acute heart failure, increased sHSP60 is also correlated with a higher risk for subsequent death/readmission for acute heart failure (Bonanad et al., 2013). Thus, sHSP60 levels could emerge as promising independent predictors of adverse cardiac events. Alternatively, exosomal HSP60 can serve as a biomarker for diagnostics, assessing prognosis, and monitoring disease progression (Bavisotto et al., 2017).

## Vascular HSP60 and Atherosclerosis

**FIGURE 2:**
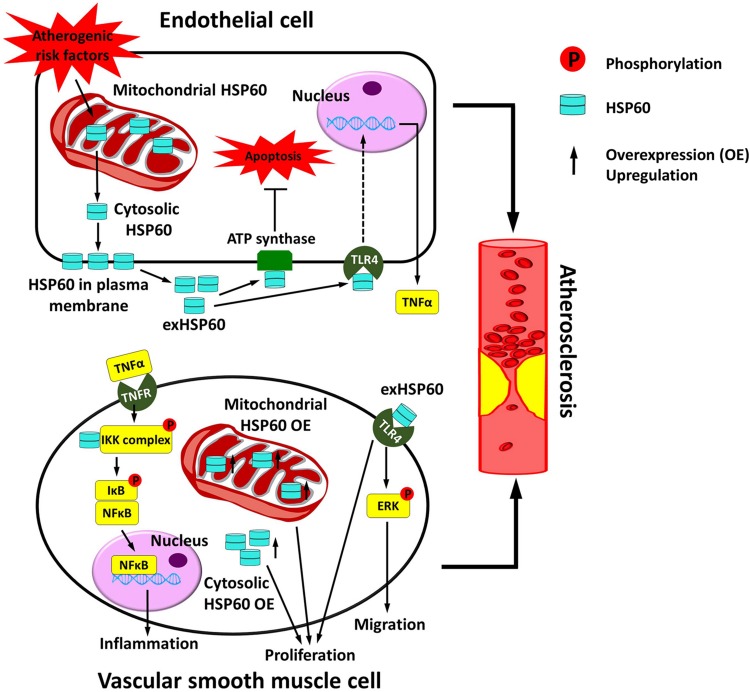
Vascular HSP60 and atherosclerosis. In endothelial cells, various atherogenic risk factors upregulate the expression of HSP60 and induce the translocation of HSP60 from the mitochondria to the cell surface and further into the cell culture supernatant. This may act as a danger signal to the atherosclerosis. exHSP60 binds to membrane ATP synthase and serves a protective role against cell apoptosis. However, exHSP60 also binds to TLR4 and induces TNFα production. In vascular smooth muscle cells (VSMCs), HSP60 is associated with the IKK complex in the cytosol and assists the phosphorylation-dependent activation of the complex upon TNFα stimulation. The activated IKK phosphorylates its substrate IκB, causes NF-κB translocation into the nucleus, and enhances transcription of the genes that are involved in inflammation. Moreover, exHSP60 and overexpression (OE) of the HSP60 with or without mitochondrial targeting peptide can induce cell proliferation. In addition, exHSP60 can bind to TLR4 to promote cell migration via activation of ERK. In short, HSP60 regulates the inflammation, proliferation and migration of VSMCs, which may further accelerate atherosclerosis.

Atherosclerosis progression is a complicated process that involves the participation of endothelial cells (ECs), VSMCs, macrophages, and other lymphocytes (Bennett et al., 2016; Gimbrone and Garcia-Cardena, 2016; Tabas and Bornfeldt, 2016). In atherosclerosis, the endothelial cell layer is disrupted by oxidized low-density lipoproteins (oxLDLs) (Negre-Salvayre et al., 2017). Macrophage foam cell formation occurs, and smooth muscle cells undergo migration and proliferation (Bennett et al., 2016). These mechanisms contribute to atherosclerotic plaque formation. It has been generally supposed that HSP60 is atherogenic (Grundtman et al., 2011). HSP60 can activate both the innate immune system via TLR4 and the adaptive immune system (Quintana and Cohen, 2011). However, the role of HSP60 in ECs and VSMCs has only been investigated in a limited number of studies. Here we describe the current understanding of how HSP60 functions on ECs and VSMCs, respectively, and discuss how vascular HSP60 is involved in the development of atherosclerosis (Figure 2).

### HSP60 May Regulate Cell Survival of ECs

Various atherogenic risk factors including physical insult (shear stress and heat), chemical stress (smoking, oxygen radicals, drugs, high sodium, and high glucose), infection (e.g., Chlamydia pneumoniae), and inflammation (LPS, inflammatory cytokines, and oxLDLs) possess the ability to regulate the expression or localization of HSP60 in ECs (Amberger et al., 1997; Hochleitner et al., 2000; Hirono et al., 2003; Wick et al., 2008, 2014; Kreutmayer et al., 2011, 2013; Mohammad and Kowluru, 2011; Zhao et al., 2015; Jakic et al., 2017). Shear stress plays a major role in the generation, progression, and destabilization of atherosclerotic plaques (Souilhol et al., 2019). Shear stress is able to induce HSP60 expression in ECs *in vitro* and *in vivo* (Hochleitner et al., 2000). ECs in atherosclerotic lesions of rabbits and humans also exhibit higher HSP60 levels than those in other regions of normal arterial intima (Kleindienst et al., 1993; Xu et al., 1994). Cigarette smoking is another significant risk factor for atherosclerosis (Knoflach et al., 2003). It has been shown that prolonged exposure to cigarette smoke alters mitochondrial structures. As a result, HSP60 is released from the mitochondria, transported to the cell surface, and then dispensed into the cell culture supernatant in ECs. Thus, cigarette smoke exposure likely accounts for the increased levels of HSP60 found in the serum of healthy young individuals exposed to second hand smoke (Bernhard et al., 2004; Kreutmayer et al., 2011). exHSP60 has been shown to induce TNFα in human umbilical vein ECs (Martinus and Goldsbury, 2018). However, there is one exceptional condition in which exHSP60 is beneficial to ECs. exHSP60 may bind to membrane ATP synthase and serve a protective role against EC acidification and cell apoptosis in the presence of anti-ATP synthase antibodies (Champagne et al., 2006; Alard et al., 2011). On the other hand, downregulation of mitochondrial HSP60 in ECs is associated with accelerated apoptosis (Mohammad and Kowluru, 2011), whereas overexpression of HSP60 in ECs may exert a protective role in digoxin-induced apoptosis (Qiu et al., 2008). A recent study using Tie2-Cre to delete HSP60 in both hematopoietic cells and ECs suggests that HSP60 may be required for embryonic erythropoiesis and vascular development (Duan et al., 2019). However, it remains to be investigated in more detail whether and how HSP60 regulates EC physiology *in vivo*.

### HSP60 May Regulate Proliferation, Survival, and Migration of VSMCs

It has been shown that the overexpression of the HSP60 with or without mitochondrial targeting peptide can induce an increase in VSMC proliferation (Hirono et al., 2003; Deniset et al., 2018). exHSP60 may also play a very important role in regulating cell proliferation of VSMCs. Recombinant human HSP60 promotes cell proliferation in venous VSMCs, which can be inhibited by the application of TLR2 and TLR4 antibodies (de Graaf et al., 2006). Interestingly, exHSP60 can also promote cell migration of VSMCs. VSMCs exposed to HSP60 exhibit increased cell migration, which is accompanied with increased expression of TLR4 and ERK activity. Knockdown of TLR4 or the use of ERK inhibitors can significantly reduce HSP60-induced VSMC migration (Zhao et al., 2015). On the other hand, cytosolic HSP60 plays an anti-apoptotic role in VSMCs. Deletion of cytosolic HSP60 in VSMCs is found to reduce the IκB kinase activation, repress the induction of NFκB-dependent survival genes, enhance apoptotic death in response to TNFα, and markedly inhibit the neointimal thickening in the balloon-injured arterial vessels (Choi et al., 2015).

### HSP60 Is Atherogenic

Under stressful states, HSP60 is translocated to the cytosol and appears on the plasma membrane and in the extracellular space. HSP60 can also be directly released from damaged and dying cells (Xu et al., 1994; Wick et al., 2014). Higher expression of HSP60 can be found in ECs in atherosclerotic lesions of rabbits and humans compared with those in other parts of normal arterial intima (Kleindienst et al., 1993; Xu et al., 1994). Consistently, elevated levels of soluble or circulating HSP60 have been shown to correlate with an increased risk of atherosclerosis (Pockley et al., 2000; Xu et al., 2000; Shamaei-Tousi et al., 2007; Grundtman et al., 2011). HSP60 on the membrane and in the extracellular space has been widely considered to act as a danger signal to the atherosclerosis in several aspects. First, HSP60 has a high degree of homology in both protein and DNA levels between different bacterial species, and from prokaryotic to eukaryotic cells (Craig et al., 1993; Grundtman et al., 2011). All humans develop cellular and humoral immunity against bacterial HSP60 either by infection or vaccination. This protective defense may increase the risk of cross-reactivity with autologous HSP60, which causes the adhesion of HSP60-reactive T cells to the endothelial cells and the initiation of the earliest inflammatory responses of atherosclerosis. In addition, antibodies against HSP60 are able to accelerate and perpetuate the disease (Schett et al., 1997; Stocker and Keaney, 2004; Grundtman et al., 2011; Wick et al., 2014). If the reversible early inflammatory stage of atherosclerosis is not interfered or risk factors remain present, irreversible late stages with atherosclerotic plaques will develop and lead to clinical consequences. Second, exHSP60 can react to TLR4 expressed in endothelial cells, smooth muscle cells, and macrophages (Xu et al., 2012), as well as activate NFκB-dependent signaling pathways. It can also promote the production of various proteolytic enzymes and cytokine such as TNF, IL10, adhesion molecules, and growth factors (Moghimpour Bijani et al., 2012). Third, exHSP60 may induce the proliferation and migration of VSMCs (Hirono et al., 2003; de Graaf et al., 2006; Zhao et al., 2015; Deniset et al., 2018), which may further accelerate atherosclerosis.

## Conclusion

It is now clear that HSP60 plays multiple regulatory roles in cardiovascular physiology. Mitochondrial HSP60 together with its chaperonin HSP10 regulates mitochondrial protein folding. Deletion of HSP60 results in mitochondrial dysfunction, chamber dilation, and heart failure. HSP60 undergoes subcellular translocation and secretion in response to stress and cell / tissue injury, and is considered as a pathogenic signal in heart failure, atherosclerosis, and other cardiovascular diseases. exHSP60 may induce cell apoptosis in cardiomyocytes and thus exacerbates the disease state of heart failure. exHSP60 may also participate in the initial step of atherosclerosis by inducing multiple inflammatory responses, and accelerates the progression of atherosclerosis by promoting VSMC cell proliferation and migration. All of these results have provided us with a better understanding of HSP60 functions in the cardiovascular system and may contribute to the future development of novel therapies targeting HSP60 for the treatment of cardiovascular disease.

## Author Contributions

YD, HT, KM, XF, ZH, and KO wrote the manuscript.

## Conflict of Interest

The authors declare that the research was conducted in the absence of any commercial or financial relationships that could be construed as a potential conflict of interest.

## References

[B1] Alard J. E. Hillion S. Guillevin L. Saraux A. Pers J. O. Youinou P. (2011). Autoantibodies to endothelial cell surface ATP synthase, the endogenous receptor for hsp60, might play a pathogenic role in vasculatides. *Plos One* 6:e14654. 10.1371/journal.pone.0014654 21326874 PMC3034716

[B2] Amberger A. Maczek C. Jurgens G. Michaelis D. Schett G. Trieb K. (1997). Co-expression of ICAM-1, VCAM-1, ELAM-1 and Hsp60 in human arterial and venous endothelial cells in response to cytokines and oxidized low-density lipoproteins. *Cell Stress Chaperones* 2 94–103. 9250400 10.1379/1466-1268(1997)002<0094:ceoive>2.3.co;2 PMC312986

[B3] Basu S. Binder R. J. Suto R. Anderson K. M. Srivastava P. K. (2000). Necrotic but not apoptotic cell death releases heat shock proteins, which deliver a partial maturation signal to dendritic cells and activate the NF-kappa B pathway. *Int. Immunol.* 12 1539–1546. 10.1093/intimm/12.11.1539 11058573

[B4] Bavisotto C. C. Cappello F. Macario A. J. L. de Macario E. C. Logozzi M. Fais S. (2017). Exosomal HSP60: a potentially useful biomarker for diagnosis, assessing prognosis, and monitoring response to treatment. *Expert Rev. Mol. Diagn.* 17 815–822. 10.1080/14737159.2017.1356230 28718351

[B5] Bennett M. R. Sinha S. Owens G. K. (2016). Vascular smooth muscle cells in atherosclerosis. *Circ. Res.* 118 692–702. 10.1161/CIRCRESAHA.115.306361 26892967 PMC4762053

[B6] Bernhard D. Huck C. W. Jakschitz T. Pfister G. Henderson B. Bonn G. K. (2004). Development and evaluation of an in vitro model for the analysis of cigarette smoke effects on cultured cells and tissues. *J. Pharmacol. Toxicol. Methods* 50 45–51. 10.1016/j.vascn.2004.01.003 15233967

[B7] Bie A. S. Comert C. Korner R. Corydon T. J. Palmfeldt J. Hipp M. S. (2020). An inventory of interactors of the human HSP60/HSP10 chaperonin in the mitochondrial matrix space. *Cell Stress Chaperones* [Epub ahead of print]. 10.1007/s12192-020-01080-6 32060690 PMC7192978

[B8] Blasi C. Kim E. Knowlton A. A. (2012). Improved metabolic control in diabetes, HSP60, and proinflammatory mediators. *Autoimmune Dis.* 2012:346501. 10.1155/2012/346501 22924123 PMC3424633

[B9] Bonanad C. Nunez J. Sanchis J. Bodi V. Chaustre F. Chillet M. (2013). Serum heat shock protein 60 in acute heart failure: a new biomarker? *Congest Heart Fail* 19 6–10. 10.1111/j.1751-7133.2012.00299.x 22702715

[B10] Campanella C. D’Anneo A. Marino Gammazza A. Caruso Bavisotto C. Barone R. Emanuele S. (2016). The histone deacetylase inhibitor SAHA induces HSP60 nitration and its extracellular release by exosomal vesicles in human lung-derived carcinoma cells. *Oncotarget* 7 28849–28867. 10.18632/oncotarget.6680 26700624 PMC5045361

[B11] Cervio E. Barile L. Moccetti T. Vassalli G. (2015). Exosomes for intramyocardial intercellular communication. *Stem Cells Int.* 2015 482171. 10.1155/2015/482171 26089917 PMC4454760

[B12] Champagne E. Martinez L. O. Collet X. Barbaras R. (2006). Ecto-F1Fo ATP synthase/F1 ATPase: metabolic and immunological functions. *Curr. Opin. Lipidol.* 17 279–284. 10.1097/01.mol.0000226120.27931.76 16680033

[B13] Chandra D. Choy G. Tang D. G. (2007). Cytosolic accumulation of HSP60 during apoptosis with or without apparent mitochondrial release: evidence that its pro-apoptotic or pro-survival functions involve differential interactions with caspase-3. *J. Biol. Chem.* 282 31289–31301. 10.1074/jbc.M702777200 17823127

[B14] Cheng M. Y. Hartl F. U. Martin J. Pollock R. A. Kalousek F. Neupert W. (1989). Mitochondrial heat-shock protein hsp60 is essential for assembly of proteins imported into yeast mitochondria. *Nature* 337 620–625. 10.1038/337620a0 2645524

[B15] Choi B. Choi M. Park C. Lee E. K. Kang D. H. Lee D. J. (2015). Cytosolic Hsp60 orchestrates the survival and inflammatory responses of vascular smooth muscle cells in injured aortic vessels. *Cardiovasc Res.* 106 498–508. 10.1093/cvr/cvv130 25870185

[B16] Christensen J. H. Nielsen M. N. Hansen J. Fuchtbauer A. Fuchtbauer E. M. West M. (2010). Inactivation of the hereditary spastic paraplegia-associated Hspd1 gene encoding the Hsp60 chaperone results in early embryonic lethality in mice. *Cell Stress Chaperones* 15 851–863. 10.1007/s12192-010-0194-x 20393889 PMC3024079

[B17] Cohen-Sfady M. Pevsner-Fischer M. Margalit R. Cohen I. R. (2009). Heat shock protein 60, via MyD88 innate signaling, protects B cells from apoptosis, spontaneous and induced. *J. Immunol.* 183 890–896. 10.4049/jimmunol.0804238 19561102

[B18] Craig E. A. Gambill B. D. Nelson R. J. (1993). Heat shock proteins: molecular chaperones of protein biogenesis. *Microbiol. Rev.* 57 402–414. 8336673 10.1128/mr.57.2.402-414.1993 PMC372916

[B19] de Graaf R. Kloppenburg G. Peter J. H. M. K. Bruggeman C. A. Stassen F. (2006). Human heat shock protein 60 stimulates vascular smooth muscle cell proliferation through Toll-like receptors 2 and 4. *Microbes Infect.* 8 1859–1865. 10.1016/j.micinf.2006.02.024 16843693

[B20] Deniset J. F. Hedley T. E. Hlavackova M. Chahine M. N. Dibrov E. O’Hara K. (2018). Heat shock protein 60 involvement in vascular smooth muscle cell proliferation. *Cell Signal.* 47 44–51. 29596871 10.1016/j.cellsig.2018.03.011

[B21] Duan Y. Wang H. Mitchell-Silbaugh K. Cai S. Fan F. Li Y. (2019). Heat shock protein 60 regulates yolk sac erythropoiesis in mice. *Cell Death Dis.* 10:766. 10.1038/s41419-019-2014-2 31601784 PMC6786998

[B22] Fan F. Duan Y. Yang F. Trexler C. Wang H. Huang L. (2019). Deletion of heat shock protein 60 in adult mouse cardiomyocytes perturbs mitochondrial protein homeostasis and causes heart failure. *Cell Death Differ.* 27 587–600. 10.1038/s41418-019-0374-x 31209364 PMC7205885

[B23] Fayet O. Ziegelhoffer T. Georgopoulos C. (1989). The groES and groEL heat shock gene products of *Escherichia coli* are essential for bacterial growth at all temperatures. *J. Bacteriol.* 171 1379–1385. 10.1128/jb.171.3.1379-1385.1989 2563997 PMC209756

[B24] Frantz S. Kobzik L. Kim Y. D. Fukazawa R. Medzhitov R. Lee R. T. (1999). Toll4 (TLR4) expression in cardiac myocytes in normal and failing myocardium. *J. Clin. Invest.* 104 271–280. 10.1172/JCI6709 10430608 PMC408420

[B25] Giannessi D. Colotti C. Maltinti M. Del Ry S. Prontera C. Turchi S. (2007). Circulating heat shock proteins and inflammatory markers in patients with idiopathic left ventricular dysfunction: their relationships with myocardial and microvascular impairment. *Cell Stress Chaperones* 12 265–274. 10.1379/csc-272.1 17915559 PMC1971236

[B26] Gimbrone M. A. Jr. Garcia-Cardena G. (2016). endothelial cell dysfunction and the pathobiology of atherosclerosis. *Circ. Res.* 118 620–636. 10.1161/CIRCRESAHA.115.306301 26892962 PMC4762052

[B27] Giricz Z. Varga Z. V. Baranyai T. Sipos P. Paloczi K. Kittel A. (2014). Cardioprotection by remote ischemic preconditioning of the rat heart is mediated by extracellular vesicles. *J. Mol. Cell Cardiol.* 68 75–78. 10.1016/j.yjmcc.2014.01.004 24440457

[B28] Go A. S. Mozaffarian D. Roger V. L. Benjamin E. J. Berry J. D. Blaha M. J. (2014). Heart disease and stroke statistics–2014 update: a report from the American heart association. *Circulation* 129 e28–e292. 10.1161/01.cir.0000441139.02102.80 24352519 PMC5408159

[B29] Grundtman C. Kreutmayer S. B. Almanzar G. Wick M. C. Wick G. (2011). Heat shock protein 60 and immune inflammatory responses in atherosclerosis. *Arterioscler. Thromb. Vasc. Biol.* 31 960–968. 10.1161/ATVBAHA.110.217877 21508342 PMC3212728

[B30] Gupta S. Knowlton A. A. (2002). Cytosolic heat shock protein 60, hypoxia, and apoptosis. *Circulation* 106 2727–2733. 10.1161/01.cir.0000038112.64503.6e 12438300

[B31] Gupta S. Knowlton A. A. (2005). HSP60, Bax, apoptosis and the heart. *J. Cell Mol. Med.* 9 51–58. 15784164 10.1111/j.1582-4934.2005.tb00336.x PMC6741334

[B32] Gupta S. Knowlton A. A. (2007). HSP60 trafficking in adult cardiac myocytes: role of the exosomal pathway. *Am. J. Physio.l Heart Circ. Physiol.* 292 H3052–H3056. 10.1152/ajpheart.01355.2006 17307989

[B33] Halcox J. P. Deanfield J. Shamaei-Tousi A. Henderson B. Steptoe A. Coates A. R. (2005). Circulating human heat shock protein 60 in the blood of healthy teenagers: a novel determinant of endothelial dysfunction and early vascular injury? *Arterioscler Thromb. Vasc. Biol.* 25:e141. 10.1161/01.ATV.0000185832.34992.ff 16258145

[B34] Hansen J. J. Durr A. Cournu-Rebeix I. Georgopoulos C. Ang D. Nielsen M. N. (2002). Hereditary spastic paraplegia SPG13 is associated with a mutation in the gene encoding the mitochondrial chaperonin Hsp60. *Am. J. Hum. Genet.* 70 1328–1332. 10.1086/339935 11898127 PMC447607

[B35] Hayoun D. Kapp T. Edri-Brami M. Ventura T. Cohen M. Avidan A. (2012). HSP60 is transported through the secretory pathway of 3-MCA-induced fibrosarcoma tumour cells and undergoes N-glycosylation. *Febs. J.* 279 2083–2095. 10.1111/j.1742-4658.2012.08594.x 22487187

[B36] Heiserman J. P. Chen L. Kim B. S. Kim S. C. Tran A. L. Siebenborn N. (2015). TLR4 mutation and HSP60-induced cell death in adult mouse cardiac myocytes. *Cell Stress Chaperones* 20 527–535. 10.1007/s12192-015-0577-0 25716072 PMC4406935

[B37] Henderson B. Fares M. A. Lund P. A. (2013). Chaperonin 60: a paradoxical, evolutionarily conserved protein family with multiple moonlighting functions. *Biol. Rev.* 88 955–987. 10.1111/brv.12037 23551966

[B38] Hirono S. Dibrov E. Hurtado C. Kostenuk A. Ducas R. Pierce G. N. (2003). Chlamydia pneumoniae stimulates proliferation of vascular smooth muscle cells through induction of endogenous heat shock protein 60. *Circ. Res.* 93 710–716. 10.1161/01.RES.0000095720.46043.F2 14500333

[B39] Hochleitner B. W. Hochleitner E. O. Obrist P. Eberl T. Amberger A. Xu Q. (2000). Fluid shear stress induces heat shock protein 60 expression in endothelial cells in vitro and in vivo. *Arterioscler. Thromb. Vasc. Biol.* 20 617–623. 10712382 10.1161/01.atv.20.3.617

[B40] Horwich A. L. Farr G. W. Fenton W. A. (2006). GroEL-GroES-mediated protein folding. *Chem. Rev.* 106 1917–1930. 10.1021/cr040435v 16683761

[B41] Jakic B. Buszko M. Cappellano G. Wick G. (2017). Elevated sodium leads to the increased expression of HSP60 and induces apoptosis in HUVECs. *Plos One* 12:e0179383. 10.1371/journal.pone.0179383 28604836 PMC5467851

[B42] Kim S. C. Stice J. P. Chen L. Jung J. S. Gupta S. Wang Y. (2009). Extracellular heat shock protein 60, cardiac myocytes, and apoptosis. *Circ. Res.* 105 1186–1195. 10.1161/CIRCRESAHA.109.209643 19875724 PMC2949276

[B43] Kirchhoff S. R. Gupta S. Knowlton A. A. (2002). Cytosolic heat shock protein 60, apoptosis, and myocardial injury. *Circulation* 105 2899–2904. 10.1161/01.cir.0000019403.35847.23 12070120

[B44] Kleindienst R. Xu Q. Willeit J. Waldenberger F. R. Weimann S. Wick G. (1993). Immunology of atherosclerosis. demonstration of heat shock protein 60 expression and T lymphocytes bearing alpha/beta or gamma/delta receptor in human atherosclerotic lesions. *Am. J. Pathol.* 142 1927–1937. 8099471 PMC1886976

[B45] Knoflach M. Kiechl S. Kind M. Said M. Sief R. Gisinger M. (2003). Cardiovascular risk factors and atherosclerosis in young males: ARMY study (Atherosclerosis Risk-Factors in Male Youngsters). *Circulation* 108 1064–1069. 10.1161/01.CIR.0000085996.95532.FF 12952827

[B46] Knowlton A. A. (2017). Paying for the tolls: the high cost of the innate immune system for the cardiac myocyte. *Adv. Exp. Med. Biol.* 1003 17–34. 10.1007/978-3-319-57613-8_2 28667552

[B47] Knowlton A. A. Gupta S. (2003). HSP60, Bax, and cardiac apoptosis. *Cardiovasc. Toxicol.* 3 263–268. 14555791 10.1385/ct:3:3:263

[B48] Knowlton A. A. Kapadia S. Torre-Amione G. Durand J. B. Bies R. Young J. (1998). Differential expression of heat shock proteins in normal and failing human hearts. *J. Mol. Cell Cardiol.* 30 811–818. 10.1006/jmcc.1998.0646 9602430

[B49] Kreutmayer S. Csordas A. Kern J. Maass V. Almanzar G. Offterdinger M. (2013). Chlamydia pneumoniae infection acts as an endothelial stressor with the potential to initiate the earliest heat shock protein 60-dependent inflammatory stage of atherosclerosis. *Cell Stress Chaperones* 18 259–268. 23192457 10.1007/s12192-012-0378-7 PMC3631098

[B50] Kreutmayer S. B. Messner B. Knoflach M. Henderson B. Niederegger H. Boeck G. (2011). Dynamics of heat shock protein 60 in endothelial cells exposed to cigarette smoke extract. *J. Mol. Cell Cardiol.* 51 777–780. 21798264 10.1016/j.yjmcc.2011.07.003 PMC3190135

[B51] Latif N. Taylor P. M. Khan M. A. Yacoub M. H. Dunn M. J. (1999). The expression of heat shock protein 60 in patients with dilated cardiomyopathy. *Basic Res. Cardiol.* 94 112–119. 10326659 10.1007/s003950050133

[B52] Lau S. Patnaik N. Sayen M. R. Mestril R. (1997). Simultaneous overexpression of two stress proteins in rat cardiomyocytes and myogenic cells confers protection against ischemia-induced injury. *Circulation* 96 2287–2294. 10.1161/01.cir.96.7.2287 9337202

[B53] Levy-Rimler G. Viitanen P. Weiss C. Sharkia R. Greenberg A. Niv A. (2001). The effect of nucleotides and mitochondrial chaperonin 10 on the structure and chaperone activity of mitochondrial chaperonin 60. *Eur. J. Biochem.* 268 3465–3472. 10.1046/j.1432-1327.2001.02243.x 11422376

[B54] Lewthwaite J. Owen N. Coates A. Henderson B. Steptoe A. (2002). Circulating human heat shock protein 60 in the plasma of British civil servants: relationship to physiological and psychosocial stress. *Circulation* 106 196–201. 10.1161/01.cir.0000021121.26290.2c 12105158

[B55] Li Y. Si R. Feng Y. Chen H. H. Zou L. Wang E. (2011). Myocardial ischemia activates an injurious innate immune signaling via cardiac heat shock protein 60 and Toll-like receptor 4. *J. Biol. Chem.* 286 31308–31319. 10.1074/jbc.M111.246124 21775438 PMC3173057

[B56] Lin K. M. Lin B. Lian I. Y. Mestril R. Scheffler I. E. Dillmann W. H. (2001). Combined and individual mitochondrial HSP60 and HSP10 expression in cardiac myocytes protects mitochondrial function and prevents apoptotic cell deaths induced by simulated ischemia-reoxygenation. *Circulation* 103 1787–1792. 11282911 10.1161/01.cir.103.13.1787

[B57] Lin L. Kim S. C. Wang Y. Gupta S. Davis B. Simon S. I. (2007). HSP60 in heart failure: abnormal distribution and role in cardiac myocyte apoptosis. *Am. J. Physiol. Heart C* 293 H2238–H2247. 10.1152/ajpheart.00740.2007 17675567

[B58] Magen D. Georgopoulos C. Bross P. Ang D. Segev Y. Goldsher D. (2008). Mitochondrial hsp60 chaperonopathy causes an autosomal-recessive neurodegenerative disorder linked to brain hypomyelination and leukodystrophy. *Am. J. Hum. Genet.* 83 30–42. 10.1016/j.ajhg.2008.05.016 18571143 PMC2443844

[B59] Magnoni R. Palmfeldt J. Christensen J. H. Sand M. Maltecca F. Corydon T. J. (2013). Late onset motoneuron disorder caused by mitochondrial Hsp60 chaperone deficiency in mice. *Neurobiol. Dis.* 54 12–23. 10.1016/j.nbd.2013.02.012 23466696

[B60] Malik Z. A. Kott K. S. Poe A. J. Kuo T. Chen L. Ferrara K. W. (2013). Cardiac myocyte exosomes: stability, HSP60, and proteomics. *Am. J. Physiol. Heart C* 304 H954–H965. 10.1152/ajpheart.00835.2012 23376832 PMC3625894

[B61] Martinus R. D. Goldsbury J. (2018). Endothelial TNF-alpha induction by Hsp60 secreted from THP-1 monocytes exposed to hyperglycaemic conditions. *Cell Stress Chaperones* 23 519–525. 10.1007/s12192-017-0858-x 29134442 PMC6045554

[B62] Meng Q. L. Li B. X. Xiao X. S. (2018). Toward developing chemical modulators of Hsp60 as potential therapeutics. *Front. Mol. Biosci.* 5:35 10.3389/fmolb.2018.00035 PMC5920047 29732373

[B63] Moghimpour Bijani F. Vallejo J. G. Rezaei N. (2012). Toll-like receptor signaling pathways in cardiovascular diseases: challenges and opportunities. *Int. Rev. Immunol.* 31 379–395. 10.3109/08830185.2012.706761 23083347

[B64] Mohammad G. Kowluru R. A. (2011). Novel role of mitochondrial matrix metalloproteinase-2 in the development of diabetic retinopathy. *Invest Ophthalmol. Vis. Sci.* 52 3832–3841. 10.1167/iovs.10-6368 21345984 PMC3109059

[B65] Negre-Salvayre A. Auge N. Camare C. Bacchetti T. Ferretti G. Salvayre R. (2017). Dual signaling evoked by oxidized LDLs in vascular cells. *Free Radic. Biol. Med.* 106 118–133. 10.1016/j.freeradbiomed.2017.02.006 28189852

[B66] Niizeki T. Takeishi Y. Watanabe T. Nitobe J. Miyashita T. Miyamoto T. (2008). Relation of serum heat shock protein 60 level to severity and prognosis in chronic heart failure secondary to ischemic or idiopathic dilated cardiomyopathy. *Am. J. Cardiol.* 102 606–610. 10.1016/j.amjcard.2008.04.030 18721521

[B67] Nisemblat S. Yaniv O. Parnas A. Frolow F. Azem A. (2015). Crystal structure of the human mitochondrial chaperonin symmetrical football complex. *Proc. Natl. Acad. Sci. U. S. A.* 112 6044–6049. 10.1073/pnas.1411718112 25918392 PMC4434751

[B68] Novo G. Cappello F. Rizzo M. Fazio G. Zambuto S. Tortorici E. (2011). Hsp60 and heme oxygenase-1 (Hsp32) in acute myocardial infarction. *Transl. Res.* 157 285–292. 10.1016/j.trsl.2011.01.003 21497776

[B69] Ohashi K. Burkart V. Flohe S. Kolb H. (2000). Cutting edge: heat shock protein 60 is a putative endogenous ligand of the toll-like receptor-4 complex. *J. Immunol.* 164 558–561. 10.4049/jimmunol.164.2.558 10623794

[B70] Ostermann J. Horwich A. L. Neupert W. Hartl F. U. (1989). Protein folding in mitochondria requires complex formation with hsp60 and ATP hydrolysis. *Nature* 341 125–130. 10.1038/341125a0 2528694

[B71] Perezgasga L. Segovia L. Zurita M. (1999). Molecular characterization of the 5’ control region and of two lethal alleles affecting the hsp60 gene in Drosophila melanogaster. *Febs Lett.* 456 269–273. 10.1016/s0014-5793(99)00963-1 10456322

[B72] Pockley A. G. Wu R. Lemne C. Kiessling R. de Faire U. Frostegard J. (2000). Circulating heat shock protein 60 is associated with early cardiovascular disease. *Hypertension* 36 303–307. 10.1161/01.hyp.36.2.303 10948094

[B73] Qiu J. Gao H. Q. Liang Y. Yu H. Zhou R. H. (2008). Comparative proteomics analysis reveals role of heat shock protein 60 in digoxin-induced toxicity in human endothelial cells. *Biochim. Biophys. Acta* 1784 1857–1864. 10.1016/j.bbapap.2008.07.006 18692161

[B74] Quintana F. J. Cohen I. R. (2011). The HSP60 immune system network. *Trends Immunol.* 32 89–95. 10.1016/j.it.2010.11.001 21145789

[B75] Rizzo M. Macario A. J. L. de Macario E. C. Gouni-Berthold I. Berthold H. K. Rini G. B. (2011). Heat shock protein-60 and risk for cardiovascular disease. *Curr. Pharm. Design.* 17 3662–3668. 10.2174/138161211798220981 22074436

[B76] Schett G. Metzler B. Kleindienst R. Moschen I. Hattmannsdorfer R. Wolf H. (1997). Salivary anti-hsp65 antibodies as a diagnostic marker for gingivitis and a possible link to atherosclerosis. *Int. Arch. Allergy Immunol.* 114 246–250. 10.1159/000237675 9363905

[B77] Shamaei-Tousi A. Stephens J. W. Bin R. Cooper J. A. Steptoe A. Coates A. R. (2006). Association between plasma levels of heat shock protein 60 and cardiovascular disease in patients with diabetes mellitus. *Eur. Heart J.* 27 1565–1570. 10.1093/eurheartj/ehl081 16762985

[B78] Shamaei-Tousi A. Steptoe A. O’Donnell K. Palmen J. Stephens J. W. Hurel S. J. (2007). Plasma heat shock protein 60 and cardiovascular disease risk: the role of psychosocial, genetic, and biological factors. *Cell Stress Chaperones* 12 384–392. 10.1379/csc-300.1 18229457 PMC2134800

[B79] Sidorik L. Kyyamova R. Bobyk V. Kapustian L. Rozhko O. Vigontina O. (2005). Molecular chaperone, HSP60, and cytochrome P450 2E1 co-expression in dilated cardiomyopathy. *Cell Biol Int.* 29 51–55. 10.1016/j.cellbi.2004.11.011 15763499

[B80] Singh B. Patel H. V. Ridley R. G. Freeman K. B. Gupta R. S. (1990). Mitochondrial import of the human chaperonin (HSP60) protein. *Biochem. Biophys. Res. Commun.* 169 391–396. 10.1016/0006-291x(90)90344-m 1972619

[B81] Souilhol C. Serbanovic-Canic J. Fragiadaki M. Chico T. J. Ridger V. Roddie H. (2019). Endothelial responses to shear stress in atherosclerosis: a novel role for developmental genes. *Nat. Rev. Cardiol.* 17 52–63. 10.1038/s41569-019-0239-5 31366922

[B82] Stocker R. Keaney J. F. Jr. (2004). Role of oxidative modifications in atherosclerosis. *Physiol. Rev.* 84 1381–1478. 10.1152/physrev.00047.2003 15383655

[B83] Tabas I. Bornfeldt K. E. (2016). Macrophage phenotype and function in different stages of atherosclerosis. *Circ. Res.* 118 653–667. 10.1161/CIRCRESAHA.115.306256 26892964 PMC4762068

[B84] Taguchi H. Makino Y. Yoshida M. (1994). Monomeric chaperonin-60 and its 50-kDa fragment possess the ability to interact with non-native proteins, to suppress aggregation, and to promote protein folding. *J. Biol. Chem.* 269 8529–8534. 7907593

[B85] Tian J. Guo X. Liu X. M. Liu L. Weng Q. F. Dong S. J. (2013). Extracellular HSP60 induces inflammation through activating and up-regulating TLRs in cardiomyocytes. *Cardiovasc. Res.* 98 391–401. 23447644 10.1093/cvr/cvt047

[B86] Wang Y. Chen L. Hagiwara N. Knowlton A. A. (2010). Regulation of heat shock protein 60 and 72 expression in the failing heart. *J. Mol. Cell Cardiol.* 48 360–366. 10.1016/j.yjmcc.2009.11.009 19945465 PMC2814075

[B87] Wick G. Jakic B. Buszko M. Wick M. C. Grundtman C. (2014). The role of heat shock proteins in atherosclerosis. *Nat. Rev. Cardiol.* 11 516–529. 25027488 10.1038/nrcardio.2014.91

[B88] Wick M. C. Mayerl C. Backovic A. van der Zee R. Jaschke W. Dietrich H. (2008). In vivo imaging of the effect of LPS on arterial endothelial cells: molecular imaging of heat shock protein 60 expression. *Cell Stress Chaperones* 13 275–285. 10.1007/s12192-008-0044-2 18465205 PMC2673942

[B89] Wiechmann K. Muller H. Konig S. Wielsch N. Svatos A. Jauch J. (2017). Mitochondrial chaperonin HSP60 is the apoptosis-related target for myrtucommulone. *Cell Chem. Biol.* 24 614.e6–623.e6. 10.1016/j.chembiol.2017.04.008 28457707

[B90] Wong S. C. Fukuchi M. Melnyk P. Rodger I. Giaid A. (1998). Induction of cyclooxygenase-2 and activation of nuclear factor-kappaB in myocardium of patients with congestive heart failure. *Circulation* 98 100–103. 10.1161/01.cir.98.2.100 9679714

[B91] Xu Q. Schett G. Perschinka H. Mayr M. Egger G. Oberhollenzer F. (2000). Serum soluble heat shock protein 60 is elevated in subjects with atherosclerosis in a general population. *Circulation* 102 14–20. 10.1161/01.cir.102.1.14 10880409

[B92] Xu Q. Schett G. Seitz C. S. Hu Y. Gupta R. S. Wick G. (1994). Surface staining and cytotoxic activity of heat-shock protein 60 antibody in stressed aortic endothelial cells. *Circ Res.* 75 1078–1085. 10.1161/01.res.75.6.1078 7525102

[B93] Xu Q. B. Metzler B. Jahangiri M. Mandal K. (2012). Molecular chaperones and heat shock proteins in atherosclerosis. *Am. J. Physiol. Heart. C* 302 H506–H514. 10.1152/ajpheart.00646.2011 PMC3353778 22058161

[B94] Zhang X. He M. Cheng L. Chen Y. Zhou L. Zeng H. (2008). Elevated heat shock protein 60 levels are associated with higher risk of coronary heart disease in Chinese. *Circulation* 118 2687–2693. 10.1161/CIRCULATIONAHA.108.781856 19106391

[B95] Zhao Y. Zhang C. X. Wei X. G. Li P. Cui Y. Qin Y. H. (2015). Heat shock protein 60 stimulates the migration of vascular smooth muscle cells via Toll-like receptor 4 and ERK MAPK activation. *Sci. Rep.* 5:15352. 10.1038/srep15352 26477505 PMC4609986

[B96] Zininga T. Ramatsui L. Shonhai A. (2018). Heat shock proteins as immunomodulants. *Molecules* 23:2846. 10.3390/molecules23112846 30388847 PMC6278532

